# *Clonorchis sinensis* adult-derived proteins elicit Th2 immune responses by regulating dendritic cells via mannose receptor

**DOI:** 10.1371/journal.pntd.0006251

**Published:** 2018-03-05

**Authors:** Lu Zhao, Mengchen Shi, Lina Zhou, Hengchang Sun, Xiaona Zhang, Lei He, Zeli Tang, Caiqin Wang, Yinjuan Wu, Tingjin Chen, Mei Shang, Xinyi Zhou, Zhipeng Lin, Xuerong Li, Xinbing Yu, Yan Huang

**Affiliations:** 1 Department of Parasitology, Zhongshan School of Medicine, Sun Yat-sen University, Guangzhou, China; 2 Key Laboratory for Tropical Diseases Control, Sun Yat-sen University, Ministry of Education, Guangzhou, Guangdong, China; 3 Provincial Engineering Technology Research Center for Diseases-vectors Control, Guangdong, Guangzhou, China; 4 Guangdong Provincial Key Laboratory of Liver Disease Research, The Third Affiliated Hospital of Sun Yat-sen University, Guangzhou, Guangdong, China; 5 Graceland Medical Center of the Sixth Affiliated Hospital of Sun Yat-sen University, Guangzhou, Guangdong, China; 6 Department of Cell Biology and Genetics, School of Pre-clinical Medicine, Guangxi Medical University, Nanning, China; Queen’s University Belfast, UNITED KINGDOM

## Abstract

**Background:**

*Clonorchis sinensis* (*C*. *sinensis*) is the most widespread human liver fluke in East Asia including China and Korea. Clonorchiasis as a neglected tropical zoonosis, leads to serious economic and public health burden in China. There are considerable evidences for an etiological relation between chronic clonorchiasis and liver fibrosis in human beings. Liver fibrosis is a highly conserved and over-protected response to hepatic tissue injury. Immune cells including CD4^+^ T cell as well as dendritic cell (DC), and pro-fibrogenic cytokines like interleukin 4 (IL-4), IL-13 have been identified as vital manipulators in liver fibrogenesis. Our previous studies had a mere glimpse of T helper type 2 (Th2) dominant immune responses as key players in liver fibrosis induced by *C*. *sinensis* infection, but little is known about the involved mechanisms in this pathological process.

**Methodology/Principal findings:**

By flow cytometry (FACS), adult-derived total proteins of *C*. *sinensis* (*Cs*TPs) down-regulated the expression of surface markers CD80, CD86 and major histocompatibility complex class II (MHC-II) on lipopolysaccharide (LPS) induced DC. ELISA results demonstrated that *Cs*TPs inhibited IL-12p70 release from LPS-treated bone marrow-derived dendritic cells (BMDC). IL-10 level increased in a time-dependent manner in LPS-treated BMDCs after incubation with *Cs*TPs. CD4^+^ T cells incubated with LPS-treated BMDCs plus *Cs*TPs could significantly elevate IL-4 level by ELISA. Meanwhile, elevated expression of pro-fibrogenic mediators including IL-13 and IL-4 were detected in a co-culture system of LPS-activated BMDCs and naive T cells containing *Cs*TPs. *In vivo*, *Cs*TPs-immunized mice enhanced expression of type 2 cytokines IL-13, IL-10 and IL-4 in both splenocytes and hepatic tissue. Exposure of BMDCs to *Cs*TPs activated expression of mannose receptor (MR) but not toll like receptor 2 (TLR2), TLR4, C-type lectin receptor DC-SIGN and Dectin-2 on the cell surface by RT-PCR and FACS. Blockade of MR almost completely reversed the capacity of *Cs*TPs to suppress LPS-induced BMDCs surface markers CD80, CD86 and MHC-II expression, and further made these BMDCs fail to induce a Th2-skewed response as well as Th2 cell-associated cytokines IL-13 and IL-4 release *in vitro*.

**Conclusions/Significance:**

Collectively, we validated that *Cs*TPs could suppress the maturation of BMDCs in the presence of LPS via binding MR, and showed that the *Cs*TPs-pulsed BMDCs actively polarized naive T helper cells to Th2 cells though the production of IL-10 instead of IL-12. *Cs*TPs endowed host with the capacity to facilitate Th2 cytokines production including IL-13 and IL-4 *in vitro* and *vivo*. The study might provide useful information for developing potential therapeutic targets against the disease.

## Author summary

In China, the morbidity of clonorchiasis resulting from the infection of *Clonorchis sinensis* (*C*. *sinensis*) increased every year and 1.5 to 2 million patients develop to the late stage—liver fibrosis, cirrhosis or cholangiocarcinoma. Proinflammatory and profibrotic cytokines produced by cells of the innate and adaptive immune systems can trigger fibroblasts and nonfibroblastic cells by transdifferentiation, especially in liver fibrosis caused by parasitic infections. T helper type 2 (Th2)-cell skewing immune responses presented during chronic clonorchiasis. However, the underlying mechanisms remain vague in Th2 immunologic cascade-related reaction following *C*. *sinensis* infection. The present study illustrated that *C*. *sinensis* adult-derived proteins (*Cs*TPs) inhibited LPS-induced maturation of bone marrow-derived dendritic cells (BMDC) via mannose receptor in vitro and led to BMDC-induced differentiation of naive T cells into Th2 cells though the production of IL-10. Our findings also confirmed that *Cs*TPs initiated Th2-cell skewing immune responses to markedly elevate the production of IL-13 and IL-4 which are closely associated with the generation of liver fibrosis.

## Introduction

Clonorchiasis, resulted from *Clonorchis sinensis* (*C*. *sinensis*) infection, is a major but surprisingly neglected public health problem in Asia, notably in China and Korea. About 15 million people are infected with *C*. *sinensis* worldwide. Among which, China has the biggest share with around 13 million people infected with the parasite. Further, the morbidity rose every year[1]. The histopathology of clonorchiasis is mainly characterized by a hyperplasia of intrahepatic bile-duct epithelium, followed by periductal and liver fibrosis in chronic cases[2]. Clinically, clonorchiasis patients show different severity of the symptoms. Some patients show only mild or unspecific symptoms, such as asthenia, nausea, indigestion, jaundice, hepatomegaly and liver tenderness. Nevertheless, chronic *C*. *sinensis* infection results in various complications in the liver and biliary systems, mainly cholelithiasis, cholangitis and cholecystitis. What’s worse, 1.5 to 2 million patients with chronic infection develop to the late stage, cirrhosis or cholangiocarcinoma[3–5].

Liver fibrosis is a reversible pathological process for excessive repair and damage of hepatic tissue that characterized by accumulation and activation of various fibroblasts, deposition of extracellular matrix (ECM) proteins including collagen. If the injury is acute or self-limited, these changes are transient. However, chronic and sustained infection, may cause considerable tissue remodeling and a progressive substitution of liver parenchyma by permanent scar tissue and subsequent cirrhosis[6–8]. Parasites represent a diverse group of pathogens that often trigger highly polarized immune responses that become tightly regulated during chronic infections[9]. Proinflammatory and profibrotic cytokines produced by cells of the innate and adaptive immune systems can trigger fibroblasts and nonfibroblastic cells by transdifferentiation, especially in liver fibrosis caused by parasitic infections[8]. In addition, numerous studies clearly point out that interferon gamma (IFN-γ) and interleukin 12 (IL-12) produced by T helper type 1 (Th1) cells have anti-fibrotic effects[10, 11]. Whereas Th2 cell is strongly pro-fibrogenic and in this setting IL-13 is acknowledged as a pivotal pro-fibrogenic mediator, since it could promote collagen production by three distinct but possibly overlapping pathways[12]. More interestingly, research shows that IL-13 is capable of stimulating collagen deposition directly and independently without the aid of transforming growth factor β 1 (TGF-β1), which is considered as the most potent pro-fibrogenic cytokine mainly produced by kupffer cells, monocytes, platelets paracrine and hepatic stellate cells [13].

Fibrosis often develops as a consequence of parasitic infections that is strongly linked with the development of a Th2 CD4^+^ T-cell response, involving IL-4 and IL-13 production[10, 14]. Th1 immune responses, which appeared during the acute phase, would shift to Th2 immune reactions accompanied by collagen deposition during long time infection of *C*. *sinensis*[15–17]. High concentrations of IgG1 in sera from mice model and patients infected with *C*. *sinensis* that suggested the dominant of Th2 immune responses[18, 19]. Our previous studies reported the markedly elevated production levels of IL-13 in the splenocytes of *C*. *sinensis*-infected BALB/c mice[15]. In addition, there are accumulating evidences disclose that parasites drive the development of Th1 or Th2 cells through their effects on dendritic cells (DC) which are the most potent antigen-presenting cells (APC)[9, 20, 21]. Th2-cell skewing immune responses presented during chronic infection of *C*. *sinensis*. However, the underlying mechanisms remain vague in Th2 immunologic cascade-related reaction following *C*. *sinensis* infection. In this study, we assessed the effects of proteins from *C*. *sinensis* on maturation and cytokines production of bone marrow-derived dendritic cells (BMDC) and subsequent influence on naive CD4^+^ T cells. In addition, we investigated the involved mechanisms.

## Methods

### Ethics statement

The conducts and procedures involving animal experiments were approved by the Animal Care and Use Committee of Sun Yat-Sen University (Permit Numbers: SYXK (Guangdong) 2010–0107). All work with animals were according to the National Institutes of Health on animal care and the ethical guidelines.

### Animals and parasites

6 to 8 weeks old female BALB/c mice were purchased from the animal center of Sun Yat-Sen University (Guangzhou, China). Mice were maintained in specific pathogen-free animal facilities with 12 h light/dark cycle and water adlibitum.

*C*. *sinensis* adults were collected from hepatobiliary ducts of *C*. *sinensis*-infected mice that were infected with 30 living *C*. *sinensis* metacercariae through intragastric administration and sacrificed at week 12.

### Preparation and purification of adult-derived total proteins of *C*. *sinensis* (*Cs*TPs)

Freshly collected *C*. *sinensis* adults were washed several times in phosphate buffered saline (PBS, PH 7.2) with penicillin and streptomycin (100 U/ml and 100 μg/ml, Gibco, USA). About 10 to 15 worms were lysed in 1ml of PBS with oscillation frequency of 30 Hz for 10 min. Supernatant containing *Cs*TPs were harvested and centrifuged at 4000 rpm for 15 min at 4 °C to remove residual tissue. *Cs*TPs were filtered through sterile 0.22 μm syringe filter and stored at -80°C until use.

### Generation and stimulation of BMDCs

BMDCs were generated from female BALB/c mice according to standard protocol[22, 23] with minor modifications as follows. Briefly, bone marrow (BM) cells were flushed from tibiae and femurs of 6 to 8 weeks old BALB/c mice with chilled RPMI-1640 medium (Gibco, USA). Red blood cells were lysed with red blood cell lysing buffer (Sigma, USA). Then, the total BM cells were counted and resuspended in 4 ml RPMI-1640 medium supplemented with 10% FBS (NQBB, Australia), 100 μg/ml streptomycin and 100 U/ml penicillin, 2.5 mM β-mercaptoethanol, 20 ng/ml mouse granulocyte-macrophage colony-stimulating factor (GM-CSF, R&D Systems, USA) and 10 ng/ml mouse IL-4 (R&D Systems, USA), then the cells were seeded in 6-well plates (Nest, China) with the density of 5 × 10^6^ cells /well. On day 3 and day 5, half culture medium was removed and 4 ml fresh RPMI-1640 medium containing the above supplements were added. Anti-mouse CD11c PerCP-Cyanine5.5 (eBioscience, USA) was used to detect the phenotypes of BM cells by flow cytometry on day 7. The immature BMDCs were collected and 1 × 10^6^ cells /well were seeded in 12-well plates in 1 ml complete RPMI-1640 medium (containing 10% FBS, 100 U/ml penicillin, and 100 μg/ml streptomycin) and pulsed with *Cs*TPs (20 μg/ml, 40 μg/ml or 80 μg/ml) or 0.5 μg/ml albumin (Alb, Fitzgerald, USA) of mouse as a control protein in the presence of 1 μg/ml lipopolysaccharide (LPS, Sigma-Aldrich, USA).

### Detection of surface markers and cytokines production of BMDCs

BMDCs were collected after pulsed for 24 h and maturation markers expressed on BMDCs surface were analyzed by flow cytometry (FACS). The following monoclonal antibodies (mAb) were used: PerCP-Cyanine5.5-conjugated anti-CD11c, FITC-conjugated anti-CD80, PE-conjugated anti-CD86, and APC-conjugated anti-MHC class II (eBioscience, USA). The cells were respectively incubated with the mAb for 30 min at 4°C in the dark, and then washed twice with PBS containing 0.5% BSA and resuspended in PBS. FACS was performed on a Beckman Coulter Gallios cytometer and analyzed by using Kaluza software (Beckman Coulter, USA). To assess IL-10 and IL-12p70 levels produced by BMDCs, the culture supernatants were centrifuged and harvested at different time points (24 h, 36 h and 48 h) after stimulation and determined by ELISA using the corresponding mouse ELISA kits (eBioscience, USA) referred to the instructions.

### Th1/Th2 cytokines expression by BMDCs and T cells co-culture

CD4^+^ T cells were isolated from spleens of BALB/c mice on the autoMACS Pro Separator by using CD4^+^ T Cell Isolation Kit (Miltenyi Biotec, Germany). 1×10^5^ isolated CD4^+^ T cells were co-cultured with 1×10^4^ BMDCs pulsed for 24 h in the round-bottomed 96-well plate (Costar, USA) in a total volume of 200 μl/well. 200 ng/ml IFN-γ (PeproTech, USA, USA), 2 ng/ml IL-12 (R&D Systems, USA) and 5 μg/ml anti-IL-4 (R&D Systems, USA) were added as Th1 controls, while 10 ng/ml IL-4 (R&D Systems, USA), 10 μg/ml anti-IL-12 and 5 μg/ml anti-IFN-γ were supplied with as Th2 controls[24]. On day 3, 10 U/ml rIL-2 (PeproTech, USA) was added and the cultures were expanded for another 7 days. After 10 days, for analysis of intracellular cytokine production, the primed CD4^+^ T cells were re-stimulated with 1× Cell Stimulation Cocktail (plus protein transport inhibitors) (eBioscience, USA) for 6 h. The cells were collected and stained with PE-Cyanine7-conjugated anti-CD3e (eBioscience, USA) and FITC-conjugated anti-CD4 (eBioscience, USA) for 30 min at 4 °C before being fixed and permeabilized with Fixation/Permeabilization buffer (eBioscience, USA) according to the manufacturer’s protocol. Finally, the cells were intracellular stained with APC-conjugated anti-IL-4 and PE-conjugated anti-IFN-γ (eBioscience, USA). FACS was performed on a Beckman Coulter Gallios cytometer and analyzed by using Kaluza software. Meanwhile, IL-13, IL-4, IL-10 and IFN-γ levels in the supernatant of culture were measured by ELISA using their suiting Mouse ELISA kits (eBioscience, USA).

Naive T cells were isolated from spleens of BALB/c mice using a CD4^+^CD62L^+^T Cell Isolation Kit II (Miltenyi Biotec, Germany) (S1E and S1F Fig). 5×10^4^ BMDCs pulsed for 24 h and 5×10^5^ naive CD4^+^ T cells were co-cultured at the conditions as mentioned above. Productions of IL-13, IL-4, IL-10 and IFN-γ in supernatants of the culture were quantified by ELISA after 10 days.

### Cytokine productions of splenocytes and liver tissue in *Cs*TPs treated mice

Female BALB/c mice were subcutaneously immunized with 100 μg of *Cs*TPs emulsified in complete Freund’s adjuvant (Sigma-Aldrich, USA) at 6 to 8 weeks of age. Mice similarly administered with an equal volume of PBS were as a negative control group (n = 15 in each group). Two booster injections were performed with 50 μg of *Cs*TPs or equal volume of PBS emulsified in incomplete Freund’s adjuvant (Sigma-Aldrich, USA) at two-week intervals.

The treated mice were sacrificed for isolation of splenocytes and hepatic tissue at 2^th^, 4^th^, 7^th^ and 10^th^ week after the first immunization, respectively. Spleens were extracted from mice and single splenocyte suspensions were isolated by using red blood cell lysing buffer (Sigma-Aldrich, USA) and 40 μm cell strainers (BD Falcon, USA). 5×10^6^/ml splenocytes were stimulated with 1× Cell Stimulation Cocktail (plus protein transport inhibitors) (eBioscience, USA) in complete RPMI-1640 medium. The supernatants were removed and the levels of IL-4, IL-10, IFN-γ and IL-13 were analyzed by ELISA after 48 h incubation.

Livers were aseptically removed from the mice and stored in TRIzol reagent (TransGen Biotech, China). Total RNAs were extracted from liver tissues following standard protocols. cDNAs were synthesized using TransScript All-in-One First-Strand cDNA Synthesis SuperMix for qPCR (One-Step gDNA Removal) kit (TransGen Biotech, China) from 1μg total RNA as manufacturer protocol described. Real-time quantitative reverse transcription polymerase chain reaction (RT-PCR) reactions were performed on CFX96 Real-Time PCR Detection System (Bio-Rad, USA) using TransStart Top/Tip Green qPCR SuperMix (TransGen Biotech, China). Specific mRNA levels of IL-4, IFN-γ, IL-10, IL-12 and IL-13 were analyzed by calculating 2^-ΔΔCt^ and normalized to a housekeeping gene (β-actin). All primers of RT-PCR were shown in Table 1.

**Table 1:** Primer sequences for quantitative real-time PCR.

Gene	Primer Sequence (5’->3’)
Mouse IL-4	
Forward primer	AGATGGATGTGCCAAACGTCCTCA
Reverse primer	AATATGCGAAGCACCTTGGAAGCC
Mouse IL-13	
Forward primer	AGCATGGTATGGAGTGTGGA
Reverse primer	TTGCAATTGGAGATGTTGGT
Mouse IL-10	
Forward primer	CAGAGCCACATGCTCCTAGA
Reverse primer	GGCAACCCAAGTAACCCTTA
Mouse IL-12	
Forward primer	GGAAGCACGGCAGCAGAAT
Reverse primer	GGCGGGTCTGGTTTGATG
Mouse IFN-γ	
Forward primer	GGCCATCAGCAACAACATAAGCGT
Reverse primer	TGGGTTGTTGACCTCAAACTTGGC
Mouse MR	
Forward primer	CTCTGTTCAGCTATTGGACGC
Reverse primer	GCTGCAACGCCGGCACCTATCAC
Mouse TLR4	
Forward primer	TCGCCTTCTTAGCAGAAACAC
Reverse primer	GCCTTAGCCTCTTCTCCTTC
Mouse TLR2	
Forward primer	CTCCTGAAGCTGTTGCGTTAC
Reverse primer	GCTCCCTTACAGGCTGAGTTC
Mouse DC-SIGN	
Forward primer	CTGCACAGTCTTCCTCTCCC
Reverse primer	TGGTACTGGGTAGATGGTTCA
Mouse Dectin-2	
Forward primer	AAGCGGAGCAGAATTTCATCA
Reverse primer	CCATTTGCCATTACCTTGTGGA

### Expression and blockade of BMDC receptors

1×10^6^ cells/ml immature BMDCs were stimulated with *Cs*TPs (20 μg/ml or 40 μg/ml) or 0.5 μg/ml Alb in the presence of 1 μg/ml LPS for 24 h. Receptors expressed on BMDCs including, toll like receptors (TLR) TLR2 and TLR4, C-type lectin receptors mannose receptor (MR), DC-SIGN and Dectin-2 were analyzed by RT-PCR. The primer sequences were listed in Table 1. MR was also assessed by FACS using FITC-conjugated anti-CD206 antibody (BioLegend, Canada). To block MR, BMDCs were incubated with 0.1 mg/ml or 1 mg/ml mannan (Absin Bioscience Inc, China) in complete RPMI-1640 medium for 30 min at 37°C prior to addition of the above indicated reagents.

### Statistical analysis

Statistical analysis was performed by programme Prism 6.0 (GraphPad Software). All data were presented as the mean values ± standard error or mean values. One-sided paired Student’s *t*-test were used to analyze differences between two experimental groups, and *P* value <0.05 was considered to be significant. Statistical analyses of the data were performed by ANOVA for multivariate analyses and only *P* value < 0.05 was considered statistically significant.

## Results

### *Cs*TPs negatively regulated the expression of surface markers on BMDCs

After being isolated from BM cells and cultured with 20 ng/ml GM-CSF and 10 ng/ml IL-4 for 7 days, more than 75% of the suspension cells and loosely adherent cells expressed CD11c, among which more than 65% did not express maturation marker CD86 by FACS (S1A and S1B Fig).

**Fig 1:**
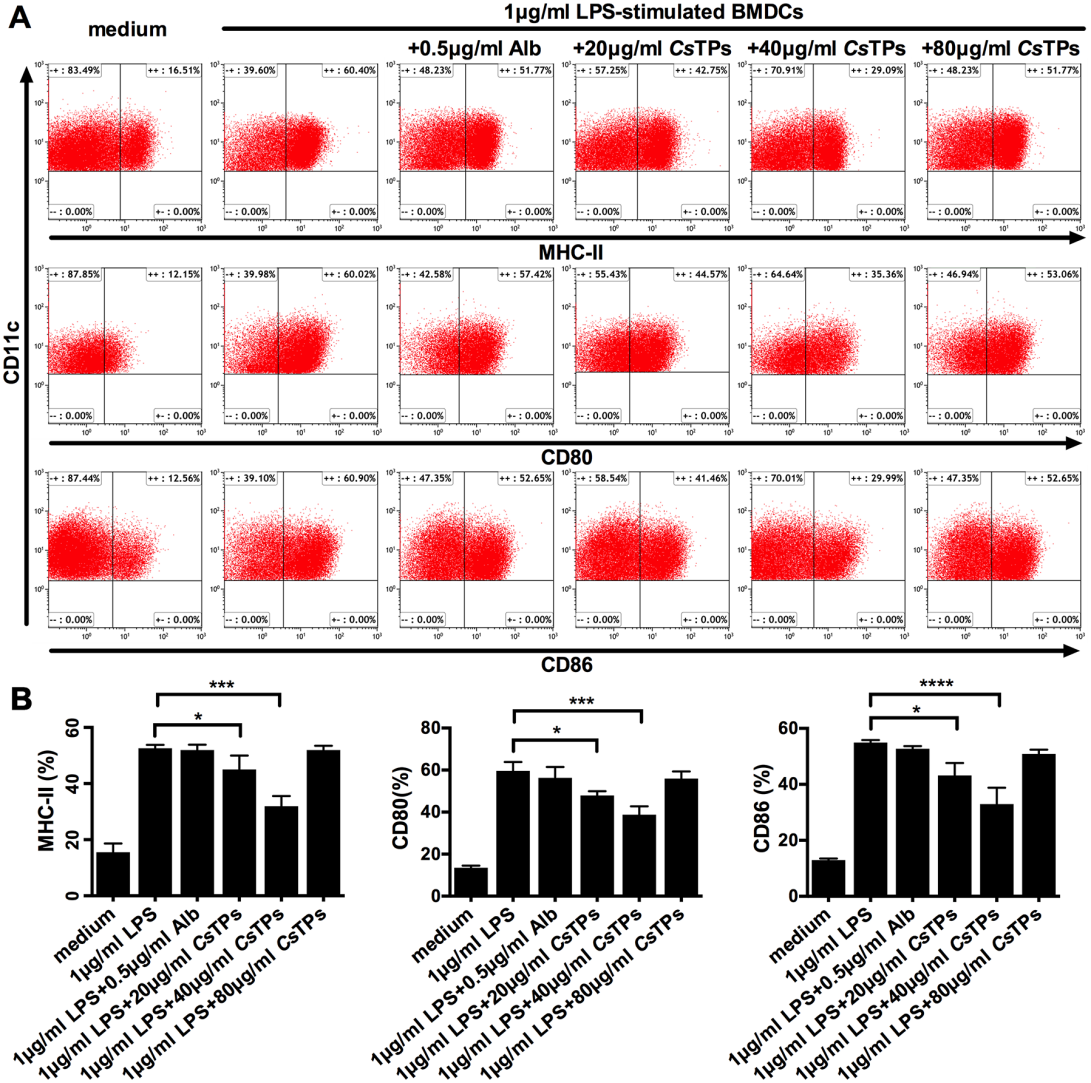
Effect of *Cs*TPs on expressions of co-stimulatory molecules on LPS-induced BMDCs. (A) Representative images of co-stimulatory molecules MHC-II, CD80 and CD86 on BMDCs detected by FACS. one marrow cells from BALB/c mice were cultured with IL-4 and GM-CSF. At Day 7, immature BMDCs were harvested and 1 × 10^6^/ml cells were treated with 20, 40 or 80 μg/ml *Cs*TPs or 0.5 μg/ml Alb (control protein) in the presence of 1μg/ml LPS as a maturation factor or medium alone for 24 h. The maturation phenotype of CD11c^+^ BMDCs were assessed by FACS for the expressions of co-stimulatory molecules MHC-II, CD80 and CD86. (B) Statistical analysis of expression percentages of the surface markers on CD11c^+^ BMDCs. Data are shown as mean ± SD of three independent experiments. Statistical significance was analyzed by one-sided paired Student’s *t*-test (*, *P* < 0.05; **, *P* < 0.01; ***, *P* < 0.001; ****, *P* < 0.0001 vs. LPS group).

The obtained BMDCs were stimulated with different concentrations of *Cs*TPs in the presence of 1 μg/m LPS. 20 μg/ml or 40 μg/ml *Cs*TPs directly suppressed the classical LPS induced up-regulation of surface markers CD80, CD86, and major histocompatibility complex class II (MHC-II) expression on BMDCs compared to the control group treated with Alb plus LPS, and the optimum concentration of *Cs*TPs was 40 μg/ml (Fig 1). As the effect of *Cs*TPs on LPS-treated BMDCs decreased obviously when the concentration up to 80 μg/ml but not in a dose-dependent manner, we identified the cytotoxic concentration of *Cs*TPs on BMDCs by CCK-8. The result illustrated that 80 μg/ml *Cs*TPs had a pronounced cytotoxic effect on BMDCs activity in the presence of LPS, while 20 μg/ml or 40 μg/ml *Cs*TPs hadn’t (S2 Fig).

### Effects of *Cs*TPs on cytokine productions of LPS-treated BMDCs

**Fig 2:**
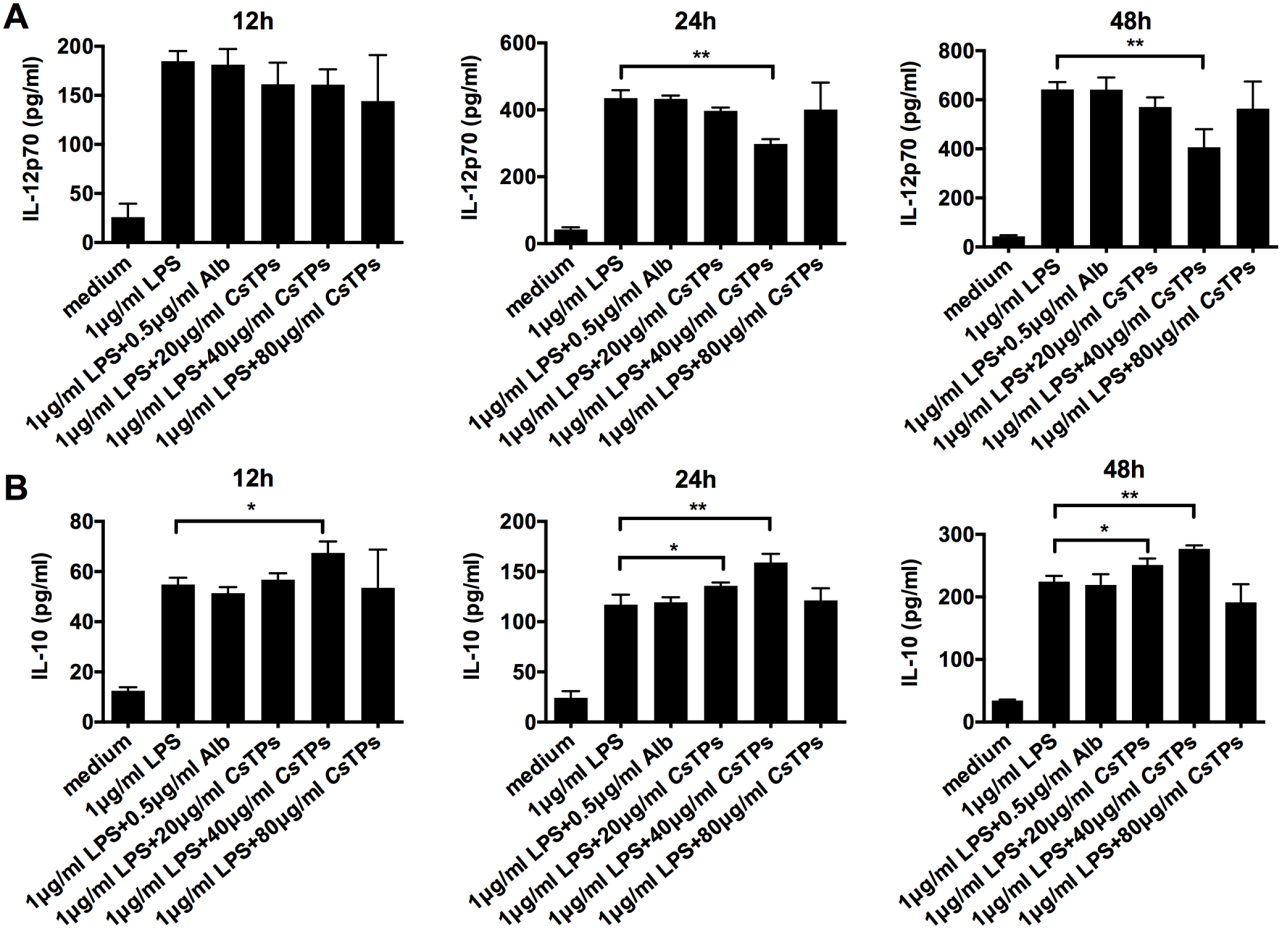
Effect of *Cs*TPs on cytokine productions of LPS-induced BMDCs. 1 × 10^6^/ml BMDCs were incubated with *Cs*TPs or Alb as indicated concentrations in the presence of LPS. Productions of IL-12p70 (A) and IL-10 (B) in the supernatants were examined by ELISA at the indicated time points (12 h, 24 h and 48 h). Results are expressed as the mean ± SD from three independent experiments (*, *P*< 0.05; **, *P* < 0.01 vs. LPS group).

DCs polarize Th cells mainly through the production of cytokines[25, 26]. ELISA results demonstrated that *Cs*TPs inhibited IL-12p70 release from LPS-treated BMDCs and the highest inhibition effect was at the concentration of 40 μg/ml (Fig 2A). IL-10 level increased in a time-dependent manner in LPS-treated BMDCs after incubation with *Cs*TPs and the optimum concentration was also 40 μg/ml (Fig 2B). IL-12p70 or IL-10 level had no statistical difference in LPS-treated BMDCs following by incubation with Alb compared to those in LPS-treated BMDCs.

### *Cs*TPs-stimulated BMDCs promoted Th2 polarization *in vitro*

**Fig 3:**
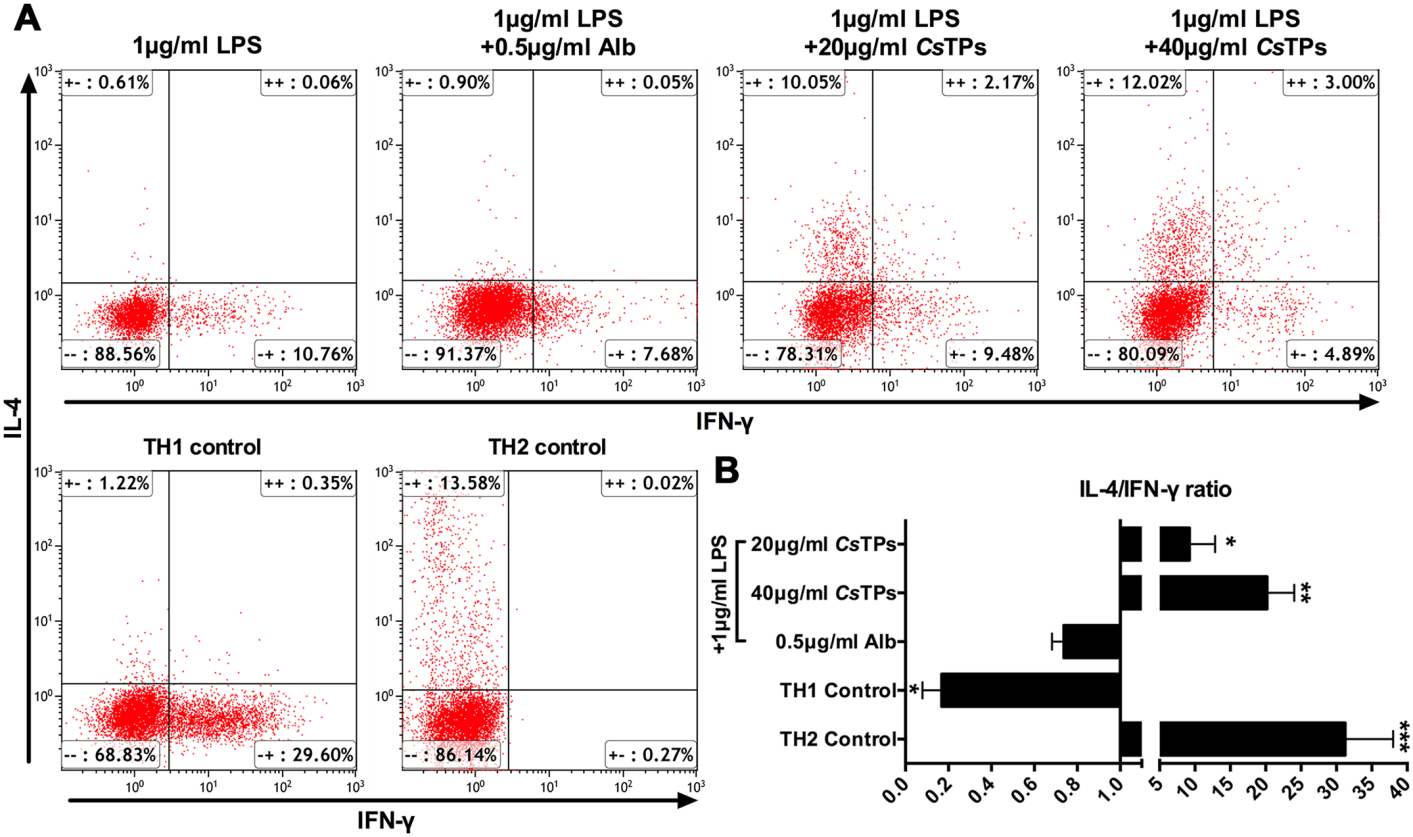
Effect of *Cs*TPs-stimulated BMDCs on the regulation of Th2 polarization. (A) Representative images of IL-4 or IFN-γ positive CD4^+^ T cells after co-culturation with *Cs*TPs-stimulated BMDCs in the presence of LPS analyzed by FACS. BMDCs were stimulated as described for 24 hours and 1×10^4^ pre-treated BMDCs were co-cultured with 1×10^5^ MACS-sorted CD4^+^ T cells for 10 d. T cells were intracellularly stained for IL-4 and IFN-γ after the stimulation of primed T cells with 1×Cell Stimulation Cocktail (plus protein transport inhibitors) for 5h, and then the stained cells were measured by FACS. (B) Statistical analysis of the ratio of IL-4 positive to IFN-γ positive CD4^+^ T cells. Three representative experiments were shown. Data are presented as mean ± SD and statistical significance was analyzed by one-sided paired Student’s *t*-test (*, *P*< 0.05; **, *P* < 0.01; ***, *P* < 0.001 vs. LPS group).

Isolated CD4^+^ T cells from BALB/c mice splenocytes (S1C and S1D Fig) were co-cultured with stimulated BMDCs at 10:1 ratio for 10 days. By intracellular cytokine staining and detecting with FACS, the ratio of IL-4 positive CD4^+^ T cells to IFN-γ positive CD4^+^ T cells in group of LPS-activated BMDC with 20 μg/ml or 40 μg/ml *Cs*TPs pulse was close to that in Th2 control group, however, the much lower ratios were showed in Th1 control group (Fig 3A and 3B).

### *Cs*TPs enhanced Th2 cytokine productions *in vitro* and *in vivo*

**Fig 4:**
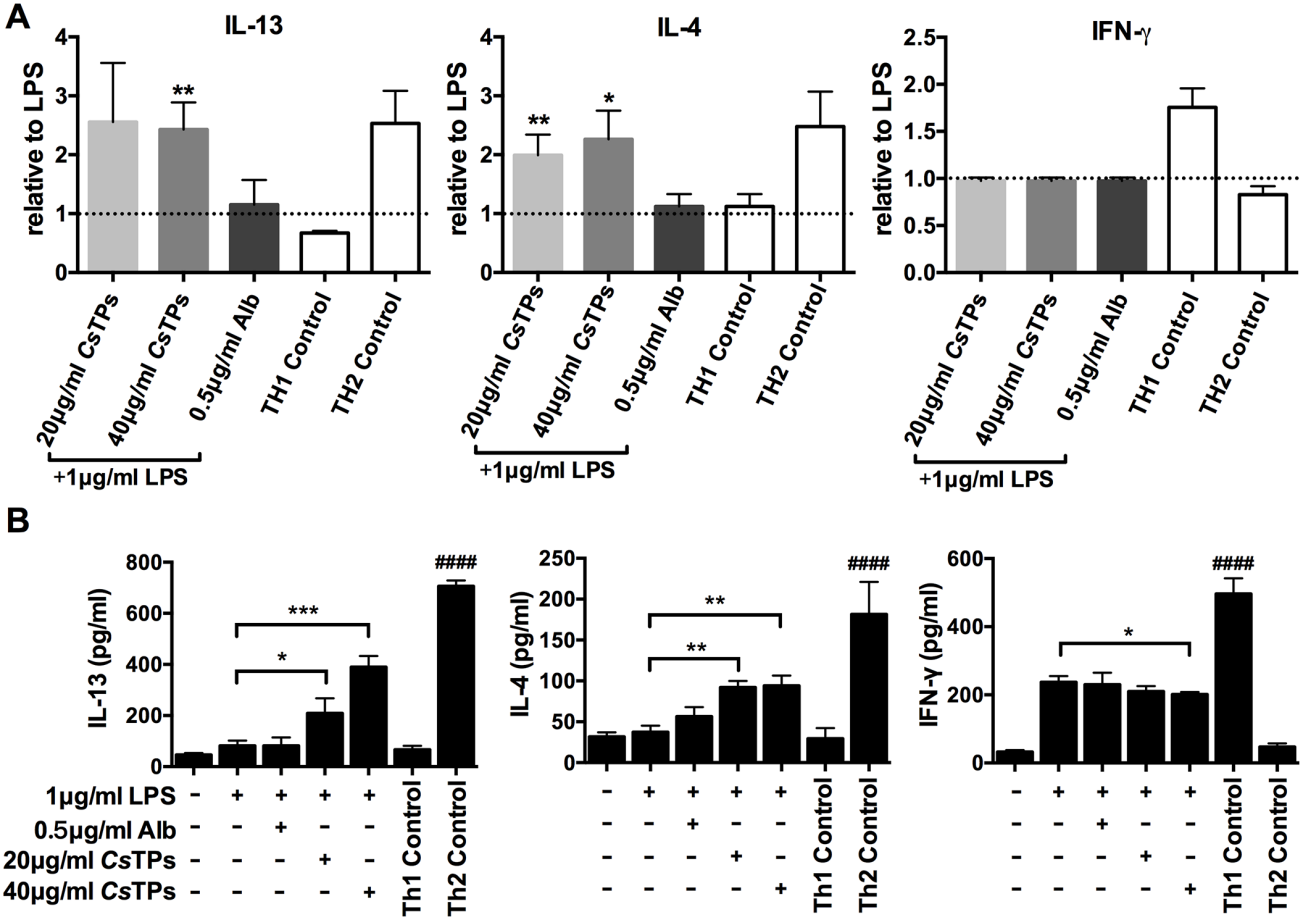
Effect of *Cs*TPs on pro-fibrotic cytokine productions in co-culture system. (A) Relative levels of IFN-γ, IL-4 and IL-13 in the supernatants of BMDCs and CD4^+^ T cells co-culture system assayed by ELISA on day 10. In different groups, LPS-activated BMDCs were respectively pulsed with 20 μg/ml *Cs*TPs, 40 μg/ml *Cs*TPs, 0.5 μg/ml Alb (control protein) or only medium. The expression level of each group was shown relative to that of LPS-stimulated BMDCs, which was set to one by a dashed line. (B) Quantification of IFN-γ, IL-4 and IL-13 levels in the supernatants of BMDCs and naïve T cells co-culture system detected on day 10. LPS-treated BMDCs (5×10^4^ /well) were incubated with 20 μg/ml *Cs*TPs, 40 μg/ml *Cs*TPs, 0.5 μg/ml Alb (control protein) or only medium for 24 h and then co-cultured with MACS-sorted naive CD4^+^ T cells from BALB/c mice splenocytes (5×10^5^/well). The supernatants were harvested and centrifuged. Cytokines production were measured by ELISA. All results are presented as mean ± SD of three independent experiments. Statistical significance was analyzed by one-sided paired Student’s *t*-test (*, *P*< 0.05; **, *P* < 0.01; ***, *P* < 0.001 vs. LPS group. ^####^, *P* < 0.0001 vs. only medium).

Th2 cytokines, mainly IL-4 and IL-13, had distinct roles in the regulation of liver fibrosis[12]. We used ELISA to examine Th1/2 cytokine productions in the co-culture system of CD4^+^T cells and *Cs*TPs-pulsed BMDCs. The secretions of IL-13 and IL-4 significantly elevated in *Cs*TPs-stimulated BMDCs group compared with those in only LPS-treated group (*P* < 0.05 or *P* < 0.01). Whereas, the productions of IL-13 and IL-4 were not influenced by Alb-treated BMDCs and there was no statistic difference in the production of IFN-γ among the groups (Fig 4A). In co-culture system of BMDCs and naive T cells, IL-13 and IL-4 levels in the supernatant of *Cs*TPs-stimulated BMDCs group were higher than those in only LPS-treated group (*P* < 0.05) by ELISA. In contrast, Alb-treated BMDCs neither drived significant IL-13 production nor IL-4 compared with those in only LPS-treated group. There was no difference of IFN-γ level among LPS alone, LPS plus Alb and LPS plus 20 μg/ml *Cs*TPs administrated groups, but LPS plus 40 μg/ml *Cs*TPs treatment negatively regulated IFN-γ level (*P* < 0.05) compared to LPS incubation (Fig 4B).

**Fig 5:**
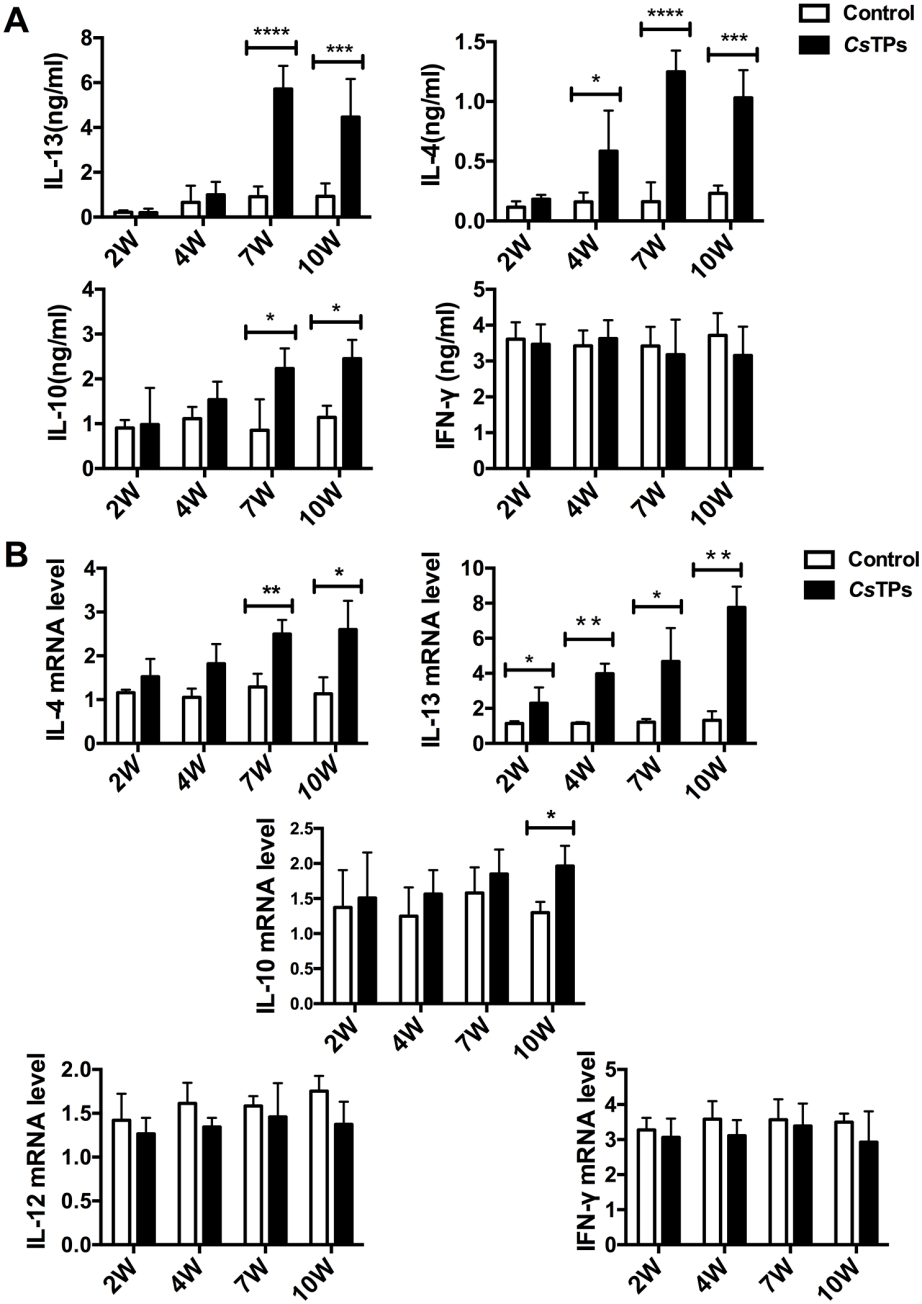
Cytokine expressions in *Cs*TPs-immunized mice. (A) Levels of IL-13, IL-4, IL-10, and IFN-γ in splenocytes of *Cs*TPs-immunized mice by ELISA. Mice were sacrificed 2, 4, 7 or 10 weeks after *Cs*TPs immunization and the splenocytes were isolated. 5 × 10^6^/ml splenocytes per well were stimulated with 1× Cell Stimulation Cocktail for 48 hours and the supernatants were analyzed. (B) mRNA levels of IL-13, IL-4, IL-10, IL-12 and IFN-γ in hepatic tissue of *Cs*TPs-immunized mice. The livers were isolated at different time points (2, 4, 7 or 10 weeks after administration). The total RNA was extracted. Specific primers were used to detect transcripts of those cytokines by RT-PCR. All data are presented as mean ± SD of triplicate wells from 3 independent experiments with 4 mice per time point per group and analyzed by calculating 2^-ΔΔCt^, normalized to a housekeeping gene (β-actin). Analysis by ANOVA indicated significant differences (*, *P* < 0.05; **, *P* < 0.01; ***, *P* < 0.001; ****, *P* < 0.0001) among the different groups.

*In vivo*, IL-13 level in splenocytes of mice immunized with *Cs*TPs increased dominantly by using ELISA compared with those from naive mice at 7 weeks (*P* < 0.0001) and 10 weeks (*P* < 0.001) post administration. IL-4 level statistically increased from 2 weeks post immunization and showed significant elevation at 7 weeks (*P* < 0.0001) and 10 weeks (*P* < 0.001) post administration. IL-10 level also statistically increased at 7 weeks (*P* < 0.05) and 10 weeks (*P* < 0.05) post treatment. There was no significant effect on the secretion of IFN-γ (Fig 5A). The mRNA levels of IL-4 and IL-13 in liver tissues of immunized mice showed distinctly enhancements with time dependence. There were statistical differences (*P* < 0.05) of IL-13 level compared with those in naive mice at all time points (2, 4, 7 and 10 weeks post treatment). IL-10 transcripts had only a marginal increase compared to those in the control group and presented a statistical elevation at 10 weeks (*P* < 0.05) post immunization. As to transcripts of IL-12 and IFN-γ, there were no statistical significant between the groups (Fig 5B).

### *Cs*TPs promoted MR expression on LPS-treated BMDCs

**Fig 6:**
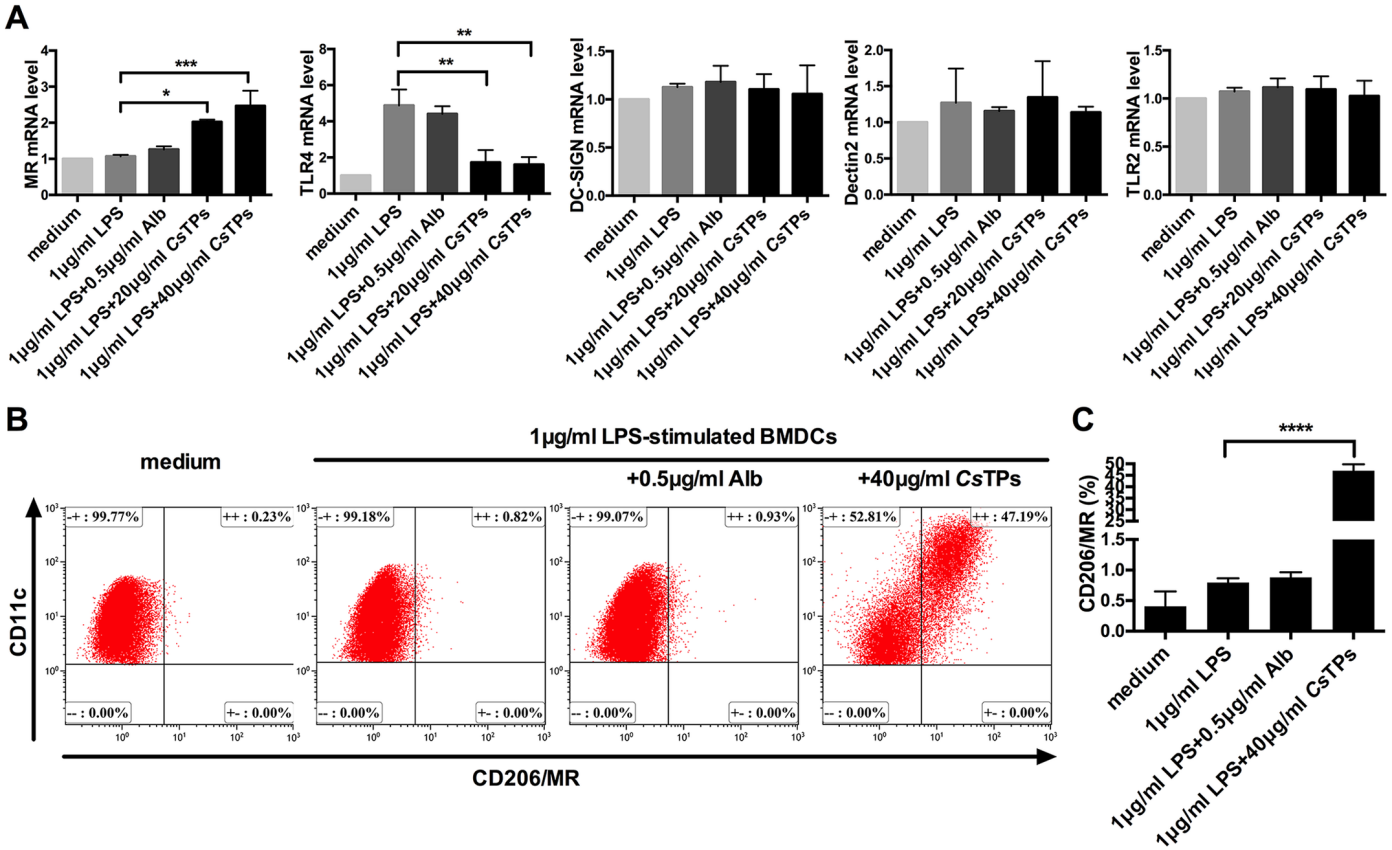
Expression of receptors on BMDCs in response to *Cs*TPs. (A) mRNA levels of MR, TLR2, TLR4, DC-SIGN and Dectin-2 on LPS-treated BMDCs after incubation with *Cs*TPs. 1 μg/ml LPS pre-treated BMDCs (2×10^6^/well) were stimulated with 20 μg/ml *Cs*TPs, 40 μg/ml *Cs*TPs or 0.5 μg/ml Alb for 24 h. The relative expression levels were examined using RT-PCR and normalized to β-actin expression. (B) Confirmation of MR expression level by FACS. BMDCs (1×10^6^/well) were stimulated with 40 μg/ml *Cs*TPs for 24 h in the presence of 1 μg/ml LPS and 0.5 μg/ml Alb as control. The expression level of MR was detected by using FITC-conjugated anti-CD206 antibody. (C) Statistical analysis of (B). All data are shown as mean ± SD of three independent experiments and statistical significance was analyzed by one-sided paired Student’s *t*-test (*, *P* < 0.05; **, *P* < 0.01; ***, *P* < 0.001; ****, *P* < 0.0001 vs. LPS group).

Pattern recognition receptors (PRRs) like TLR-2, DC-SIGN (CD209), Dectin-2 and MR (CD206) on DC are documented to be related to a more Th2-skewing response[27–30]. Transcripts of TLR2, DC-SIGN, Dectin-2 and MR on BMDCs were detected by RT-PCR to sift the specific receptor through which *C*sTPs triggered BMDC-induced polarization of Th2 cell. MR transcripts remarkably increased on LPS plus *Cs*TPs stimulated BMDCs compared to those on only LPS-treated BMDCs (*P* < 0.05), but not TLR2, Dectin-2 or DC-SIGN (Fig 6A). Conversely, the high expression of TLR4 mRNA had been observed on LPS-stimulated BMDCs as Th1 response control. For further verification, we used FACS to examine the MR expression. It was verified that 40 μg/ml *Cs*TPs could activate near fifty percent MR on the surface of LPS-activated BMDCs by FACS in contrast to those of only LPS group (*P* < 0.0001), however, Alb did not obviously interfere with the expression of MR on LPS-pulsed BMDCs (Fig 6B and 6C).

### Effects of mannan as an inhibitor of MR on expression of surface markers of BMDCs and cytokine production in co-culture system

**Fig 7:**
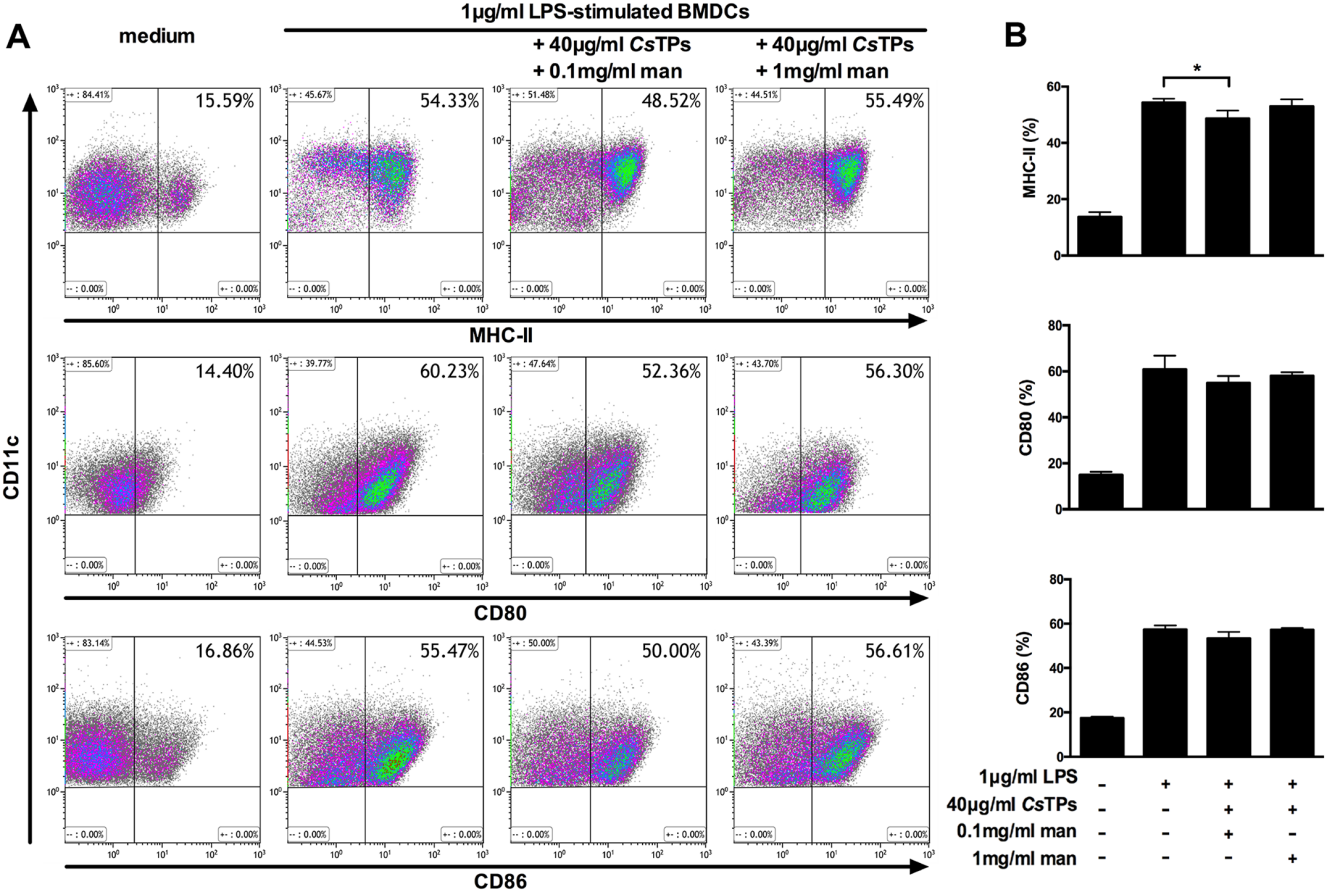
Effects of mannan as an inhibitor of MR on expression of surface markers of BMDCs. (A) Representative images of FACS. 1 × 10^6^/ml LPS-treated BMDCs were pre-incubated with 0.1 mg/ml or 1 mg/ml mannan for 30 min prior to addition of 40 μg/ml *Cs*TPs for 24 h. The expression levels of maturation markers MHC-II, CD80 and CD86 on CD11c^+^ BMDCs were assessed. (B) Statistical analysis of the expression percentages of surface markers on BMDCs. Data are shown as mean ± SD of three independent experiments. Statistical significance was analyzed by one-sided paired Student’s *t*-test (*, *P* < 0.05 vs. LPS group).

**Fig 8:**
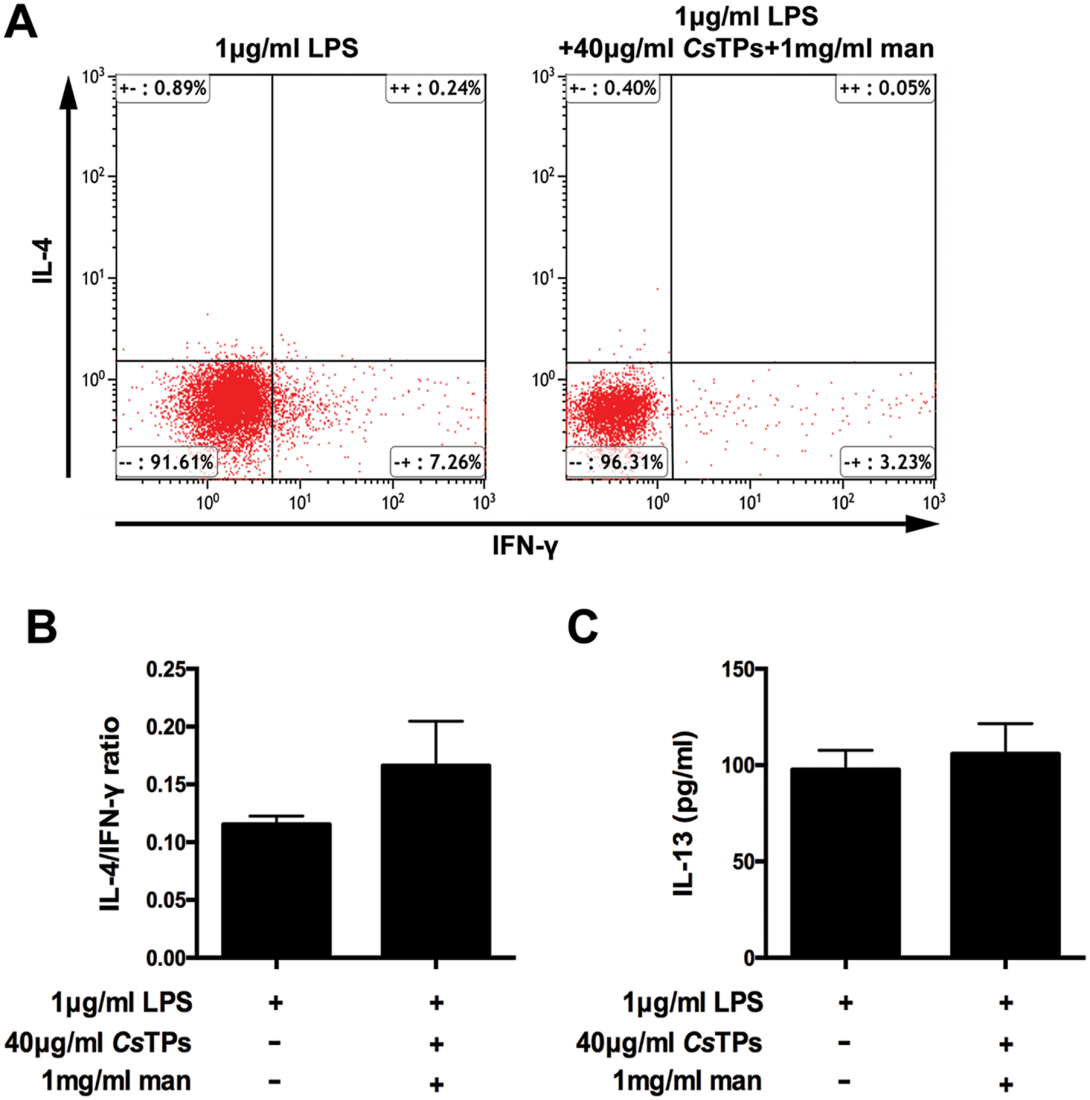
Influence of mannan as an inhibitor of MR on cytokine productions in co-culture system of BMDCs and CD4^+^ T cells. (A) Percentages of IL-4 positive or IFN-γ positive CD4^+^ T cells detected by FACS. After 30 min pre-incubation with 1 mg/ml mannan, BMDCs were stimulated with 1 μg/ml LPS and 40 μg/ml *Cs*TPs for 24 hours. 1×10^4^ pre-treated BMDCs were then co-cultured with 1×10^5^ MACS-sorted CD4^+^ T cells for 10 d. The T cells were intracellular stained with APC-conjugated anti-IL-4 and PE-conjugated anti-IFN-γ. Representative results were shown as dot plots. (B)Statistical analysis of the ratio of single positive IL-4^+^ or IFN-γ^+^ CD4^+^ T cells. (C) Measurement of IL-13 Level by using ELISA. The supernatants were harvested from the above co-culture system after 10 d. IL-13 level was assayed. These results are shown as mean ± SD of three independent experiments. Statistical significance was analyzed by one-sided paired Student’s *t*-test (no significant difference, *P*>0.05 vs. LPS group).

Soluble mannan was used as a MR blocker via competitive inhibition. There was no obvious difference of MHC-II, CD80 or CD86 expression among groups of LPS-activated BMDCs with 1 mg/ml mannan plus 40 μg/ml *Cs*TPs, LPS-activated BMDCs and medium by FACS. No statistical difference in the percentage of cells expressed CD80 or CD86 was observed among groups of LPS-activated BMDCs with 0.1 mg/ml mannan plus 40 μg/ml *Cs*TPs, LPS-activated BMDCs and medium (Fig 7A and 7B). In co-culture system of BMDCs and CD4^+^ T cells, there was no statistical difference in the ratio of IL-4 positive CD4^+^ T cells to IFN-γ positive CD4^+^ T cells between the groups of LPS-treated BMDCs and BMDCs pretreated with LPS and 1 mg/ml mannan by 40 μg/ml *Cs*TPs pulse by FACS (Fig 8A and 8B). IL-13 level in the supernatants of the groups showed no significant difference (Fig 8C).

## Discussion

Parasites always activate greatly polarized immune responses, especially during chronic infection. Our previous studies had also confirmed that compare with mechanical obstruction of the worm, the regulation of host immune responses was triggered much earlier and more important in liver fibrosis caused by a chronic infection with *C*. *sinensis*. Infection with *C*. *sinensis* has been demonstrated to promote the generation of liver fibrosis by eliciting Th2 immune response of the host[15, 16, 31]. Nevertheless, the immune regulatory pathway that could contribute to the pathological processes are currently not well known. It has been demonstrated that DC is pivotal for the recognition of helminth antigens as well as plays an essential role in regulating immune responses, in particular, priming initial T cell[9, 18, 32–35]. DC is increasingly recognized as a key mediator for the direction of Th1/Th2 polarization, which is closely related to the mature situation of DC. Mature DC is mainly characterized by the up-regulation of co-stimulatory molecules CD80 and CD86 and the translocation of MHC molecules such as MHC-II to the cell surface[36]. Antigens from parasites are able to induce maturation of DC mostly via TLRs pathway. The mature DC polarizes Th1 responses though the production of IL-12 to contribute to liver inflammation as well as play a protective role against to fibrosis[7, 37]. In contrast to mature DC, immature DC as a consequence of a fail to classically mature when exposed to antigens derived from parasitic helminthes, does not up-regulate surface molecules such as MHC-II, CD80 and CD86. And immature DC has also been found to have the ability to present antigen to CD4^+^ T cells and involves in triggering Th2 responses by the production of IL-10[38, 39]. Meanwhile, immature DC cloud be distinguished by their low production of IL-12, which is also thought to be a prerequisite for their Th2-inducing capacity[40]. We had previously identified that a recombinant protein from *C*. *sinensis* could promote Th2 response during the chronic infection via modulating DC maturation, as well as production of IL-12p70 and IL-10[18]. In this study, we showed that natural *Cs*TPs suppressed the classical LPS-induced BMDC maturation by significantly reducing the expression of CD80, CD86, and MHC-II (Fig 1A and 1B). These results were consistent with the function that *Cs*TP has been observed in allergic airway inflammation, as a previous study showed that *Cs*TP interfered with the ability of airway DC to initiate initial T cells in draining lymph nodes (dLN) by restraining the secretion of CD80, CD86 and CD40 in LPS or ovalbumin (OVA)-stimulated DC[41].

It is well known that DC is crucial to the differentiation of CD4^+^ T cell. Th2 cell as pro-fibrogenic cell has such potential to contribute for liver fibrosis by its effect on type 2 immune response[6, 12]. We therefore speculated that in *C*. *sinensis*-induced liver fibrosis, the modulation of *Cs*TPs-induced DC might be the initiation of the subsequent immunologic cascade as its strong capacity for priming type 2 immune response and CD4^+^ T cell has a crucial role in orchestrating this immune response. Indeed, ample evidences determine that the relative balance of Th1 and Th2 immune response has been recognized as a key mediator for regulating the reversible pathological process between infectious disease-induced liver inflammation and liver fibrosis. The co-administration of the Th1 cell cytokine IL-12 with *Schistosoma spp*. decreased the granuloma formation and markedly reduced the fibrosis that are associated with this infection[10]. Our results showed a diminished expression of IL-12 that could prevent the generation of Th1-polarized responses. Therefore, *Cs*TPs as strongly pro-fibrogenic antigens have been demonstrated from the opposite angle. Moreover, we found that *Cs*TPs-stimulated BMDC potently triggered the differentiation of T cell toward to a Th2 cell profile (Fig 2A and 2B). The secretion of IL-13 dramatically increased from a co-culture system of *Cs*TPs-stimulated BMDCs and naive T cells (Fig 2D). Our previous research suggested that *C*. *sinensis*-infected mice could induce Th2 immune response by expressing markedly increased levels of Th2 cytokine IL-4 and IL-13[15, 16] and promote early inflammatory cell infiltration while dense collagen deposition over time in hepatic tissue[15]. In this study, IL-4, IL-13 and IL-10 not IFN-γ were expressed in a high percentage of splenocytes and hepatic tissue in *Cs*TPs-immunized mice too compared with the control groups (Fig 3A and 3B). The results suggested that *Cs*TPs were of great immunogenicity and could strongly drive the Th2-type immune responses, especially promote the expression of IL-13 both *in vitro* and *in vivo*. As IL-13 is considered the major pro-fibrotic mediator[12], we speculated that *Cs*TPs-induced high level of IL-13 may contribute primarily to the generation and development of liver fibrosis caused by *C*. *sinensis* infection.

A large body of evidences attest to the fact that the activation of specific receptors on DC can promote Th2 responses[25, 36]. Several receptors on DC, in particular, TLR and CLR that could bind with antigens derived from helminths which are considered to be Th2 stimuli have been identified, including soluble tachyzoite antigens of *Toxoplasma gondii* binding to MyD88-induced TLR[42], Lewis-x derived from soluable egg antigens of *Schistosoma mansoni* binding to DC-SIGN[43], lipophosphoglycan of *Leishmania mexicana* binding to DC-SIGN[44, 45]and glycosylated *Schistosoma mansoni* omega-1 binding to MR[30]. In this study, we screened out MR on BMDC from a number of pattern recognition receptors (TLR-2, DC-SIGN, Dectin-2 and MR) that had been regarded as the potential elements to direct Th2 responses, and identified that it was the specific receptor to *Cs*TPs (Fig 4). Studies about schistosome indicated the roles of MR in recognizing glycosylated antigens and initiating Th2 immune responses at different stages of the infection [28][46]. MR, which has extensively been studied in DC, is a member of type I C-type lectin receptor superfamily of homologous proteins and binds glycans in a calcium-dependent manner[47]. MR has a great effect on recognizing and endocytosing variety pathogens including parasites and has been considered as a pattern recognition receptor involved in host immunity. In mice, immature DC are activated via the TLR-4 ligand LPS to become mature DC by up-regulate the costimulatory molecules CD80, CD86 and MHC-II[48, 49]. We found that so simultaneously with the high expression of MR, *Cs*TPs suppressed the production of TLR4 that was stimulated with LPS (Fig 4A). Besides, the blockade of MR with soluble mannan significantly impaired the inhibitory effect on expression of CD80, CD86, and MHC-II by *Cs*TPs (Fig 5A and 5B). It illustrated that an absence of MR on *Cs*TPs-induced BMDC were neither able to polarize Th2 effectors (Fig 5C and 5D), nor promote the secretion of IL-13 (Fig 5E). Thus, MR might have a pivotal impact on the ability of DC to regulate pro-fibrogenic cytokine via inducing Th2 polarization. That how *Cs*TPs-MR interaction contributes to Th2-type immune responses awaited further researches. In addition, highly glycosylated soluble antigens are generally responsible for Th2 polarization via MR, so that glycoprotein from *Cs*TPs that could bind to MR and subsequent modulate DC function remains to be identified.

In summary, we validated *in vitro* that *Cs*TPs could suppress the maturation of BMDCs in the presence of LPS via binding MR, and showed that the *Cs*TPs-pulsed BMDCs actively polarized naive T helper cells to Th2 cells though the production of IL-10 instead of IL-12. Our findings also illustrated that *Cs*TPs endowed host with the capacity to facilitate a Th2 cytokine production including IL-13 and IL-4 *in vitro* and *vivo*, thus possibly promoting the formation and development of liver fibrosis. Our study underscored a crucial role of *Cs*TPs in immune responses and liver fibrosis during infection of *C*. *sinensis*, which might provide useful information for developing potential therapeutic targets against the disease.

## Supporting information

S1 Fig Identification of BMDCs, CD4^+^ T cells and naive CD4^+^ T cells. (A) Generation of BMDCs from BM cells after 7 d culture with 20 ng/ml GM-CSF and 10 ng/ml IL-4. CD11c^+^ cells were detected by FACS. More than 75% of the suspension cells expressed CD11c. (B) Immaturity of BMDCs unstimulated with LPS. The expression level of maturation marker CD86 on above CD11c^+^ BMDCs were assessed by FACS. More than 65% of the BMDCs generated from BM cells were immature BMDCs. (C) & (D) CD4^+^ T cells were isolated from single-cell suspension using the CD4^+^ T Cell Isolation Kit by MACS. (C) The expression levels of CD3 and CD4 on spleen lymphocytes before sorting were assessed by FACS. (D) CD4^+^ T cells after depletion of non-CD4^+^ T cells were assessed by FACS. More than 94% of the obtained cells were CD3^+^CD4^+^ T cells. (E) & (F) Naive CD4^+^ T helper cells from the suspensions of mouse spleen cells. (E) The percentage of CD3^+^CD4^+^CD62L^+^CD44^-^ cells from spleen lymphocytes before sorting were assessed by FACS. (F) Naive CD4^+^ T cells were isolated from a single-cell suspension from mouse spleen using the Naive CD4^+^ T Cell Isolation Kit by MACS. More than 78% of the separated cells were CD3^+^CD4^+^CD62L^+^CD44^-^ cells.
(TIF)

S2 Fig Cytotoxic effect of *Cs*TPs on BMDCs. The Cell Counting Kit-8 (CCK-8) assay was used to determine cytotoxic effect of *Cs*TPs on BMDCs by testing the number of survival cells. 1×10^5^ /well BMDCs were added to a 96-well plate (Nest, China) in 100 μl complete RPMI-1640 medium per well and pulsed with or without 1μg/ml LPS and different concentrations of *Cs*TPs (20 μg/ml, 40 μg/ml or 80 μg/ml) for 24 h. 10 μl CCK-8 reagent (Dojindo, Japan) was then added to each well. After 2 h of incubation, the absorbance of each well was tested at 450 nm (BioTek, USA). All data are presented as mean ± SD sextuplicate wells of 3 independent experiments and statistical significance was analyzed by one-sided paired Student’s *t*-test (*, *P*< 0.05; **, *P* < 0.01; ***, *P* < 0.001 vs. LPS group).
(TIF)
