# Supplementary figures and images for: Clonorchis sinensis adult-derived proteins elicit Th2 immune responses by regulating dendritic cells via mannose receptor
Source: PLoS Negl Trop Dis. 2018 Mar 5;12(3):e0006251. doi: 10.1371/journal.pntd.0006251 (PMC5854424; doi:10.1371/journal.pntd.0006251)

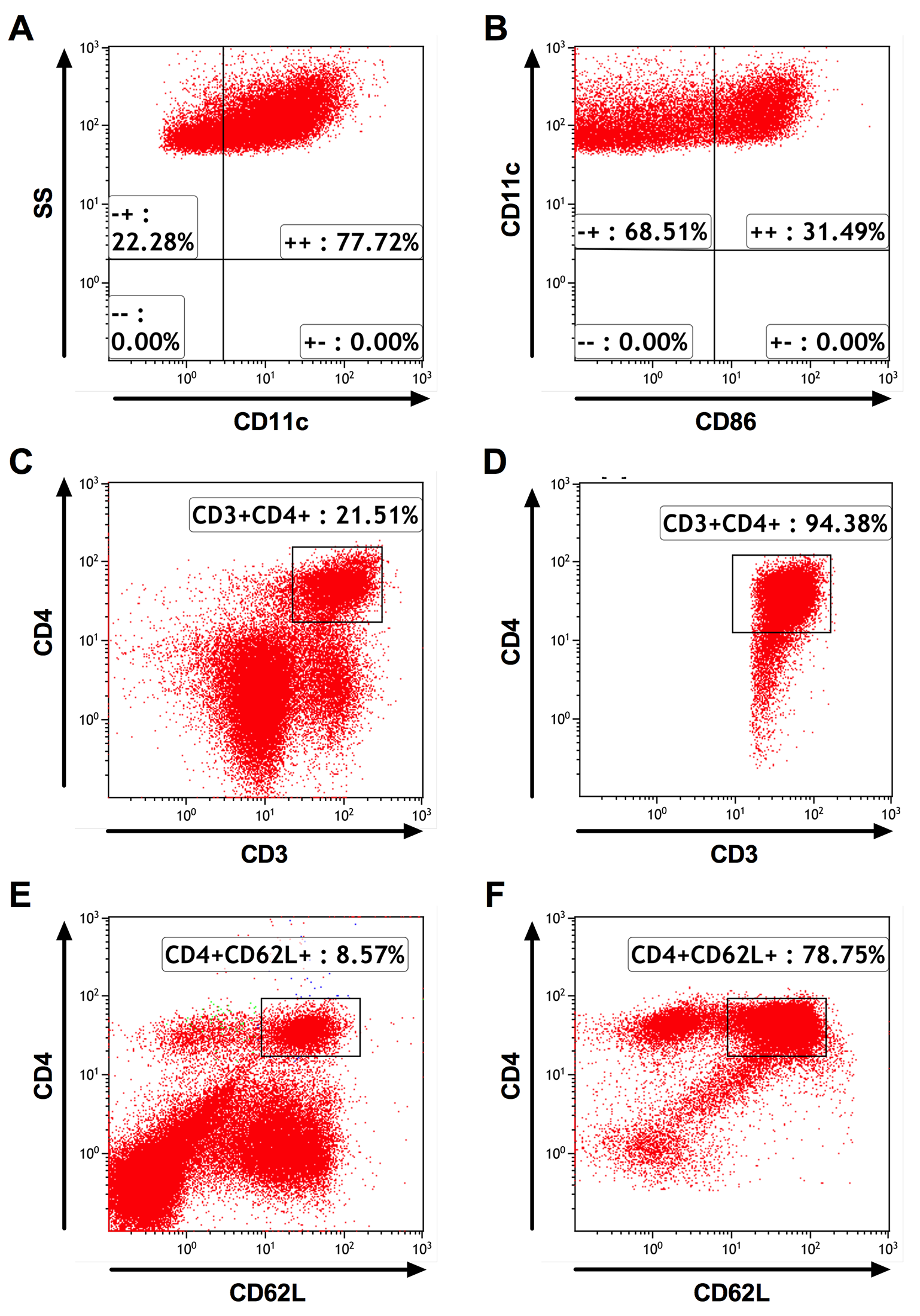

Supplement: S1 Fig — (A) Generation of BMDCs from BM cells after 7 d culture with 20 ng/ml GM-CSF and 10 ng/ml IL-4. CD11c+ cells were detected by FACS. More than 75% of the suspension cells expressed CD11c. (B) Immaturity of BMDCs unstimulated with LPS. The expression level of maturation marker CD86 on above CD11c+ BMDCs were assessed by FACS. More than 65% of the BMDCs generated from BM cells were immature BMDCs. (C) & (D) CD4+ T cells were isolated from single-cell suspension using the CD4+ T Cell Isolation Kit by MACS. (C) The expression levels of CD3 and CD4 on spleen lymphocytes before sorting were assessed by FACS. (D) CD4+ T cells after depletion of non-CD4+ T cells were assessed by FACS. More than 94% of the obtained cells were CD3+CD4+ T cells. (E) & (F) Naive CD4+ T helper cells from the suspensions of mouse spleen cells. (E) The percentage of CD3+CD4+CD62L+CD44- cells from spleen lymphocytes before sorting were assessed by FACS. (F) Naive CD4+ T cells were isolated from a single-cell suspension from mouse spleen using the Naive CD4+ T Cell Isolation Kit by MACS. More than 78% of the separated cells were CD3+CD4+CD62L+CD44- cells. (TIF) [file pntd.0006251.s001.tif]

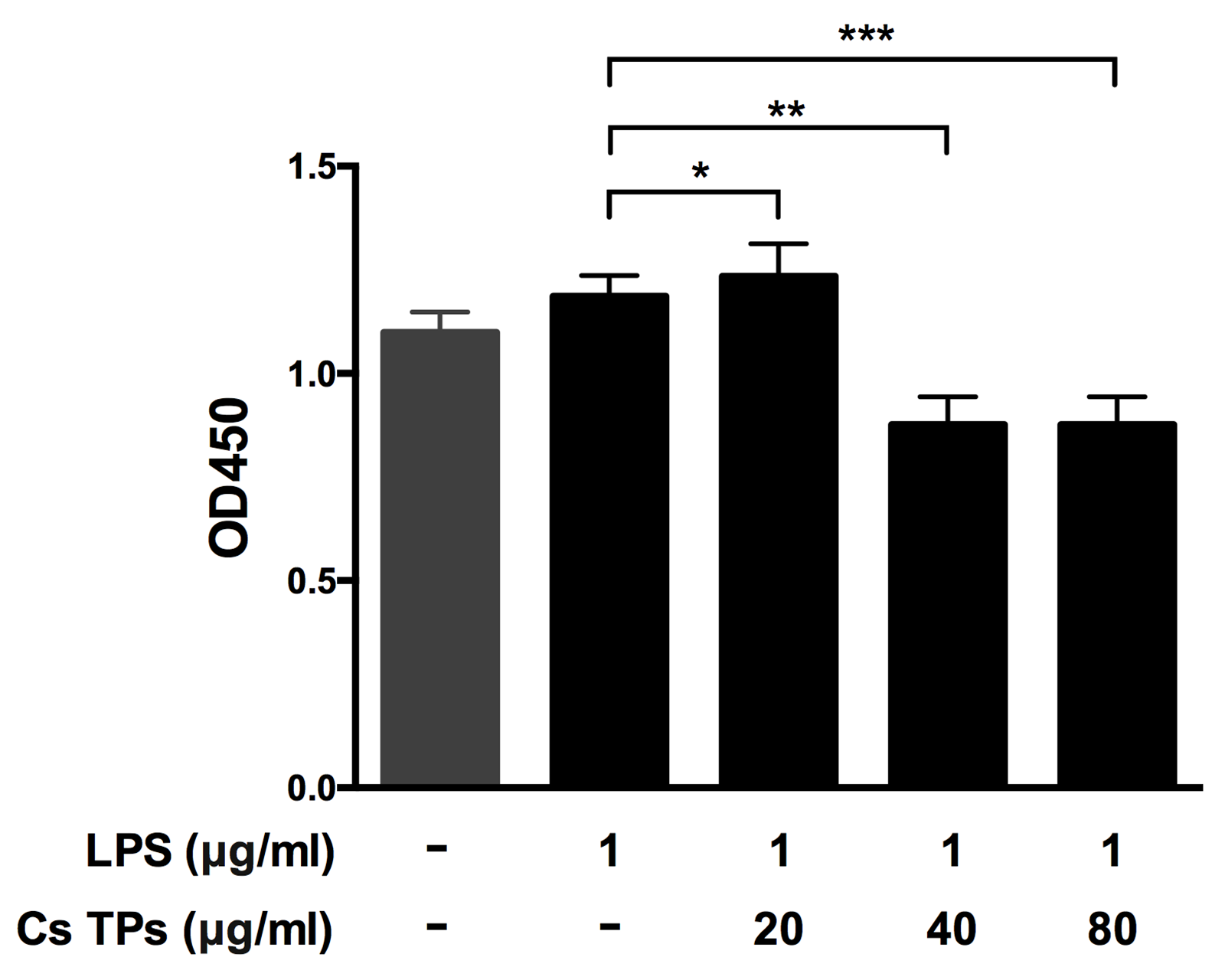

Supplement: S2 Fig — The Cell Counting Kit-8 (CCK-8) assay was used to determine cytotoxic effect of CsTPs on BMDCs by testing the number of survival cells. 1×10^5^ /well BMDCs were added to a 96-well plate (Nest, China) in 100 μl complete RPMI-1640 medium per well and pulsed with or without 1μg/ml LPS and different concentrations of CsTPs (20 μg/ml, 40 μg/ml or 80 μg/ml) for 24 h. 10 μl CCK-8 reagent (Dojindo, Japan) was then added to each well. After 2 h of incubation, the absorbance of each well was tested at 450 nm (BioTek, USA). All data are presented as mean ± SD sextuplicate wells of 3 independent experiments and statistical significance was analyzed by one-sided paired Student’s t-test (*, P< 0.05; **, P < 0.01; ***, P < 0.001 vs. LPS group). (TIF) [file pntd.0006251.s002.tif]
